# Malignant mesotheliomas in former miners and millers of crocidolite at Wittenoom (Western Australia) after more than 50 years follow-up

**DOI:** 10.1038/bjc.2012.23

**Published:** 2012-02-07

**Authors:** G Berry, A Reid, P Aboagye-Sarfo, N H de Klerk, N J Olsen, E Merler, P Franklin, A W Musk

**Affiliations:** 1 Sydney School of Public Health, University of Sydney, Sydney, New South Wales 2006, Australia; 2 Centre for Medical Research, University of Western Australia, Perth, Western Australia, Australia; 3 School of Population Health, University of Western Australia, Perth, Western Australia, Australia; 4 Centre for Child Health Research, University of Western Australia, Perth, Western Australia, Australia; 5 Veneto Mesothelioma Registry Occupational Health Unit, Local Health Authority, National Health Service, Padua, Italy; 6 Sir Charles Gairdner Hospital, Nedlands, Western Australia 6009, Australia

**Keywords:** crocidolite, asbestos, mesothelioma, predictions, Wittenoom

## Abstract

**Background:**

To report the number of malignant pleural and peritoneal mesotheliomas that have occurred in former Wittenoom crocidolite workers to the end of 2008, to compare this with earlier predictions, and to relate the mesothelioma rate to amount of exposure.

**Methods:**

A group of 6489 men and 419 women who had worked for the company operating the former Wittenoom crocidolite mine and mill at some time between 1943 and 1966 have been followed up throughout Australia and Italy to the end of 2008.

**Results:**

The cumulative number of mesotheliomas up to 2008 was 316 in men (268 pleural, 48 peritoneal) and 13 (all pleural) in women. There had been 302 deaths with mesothelioma in men and 13 in women, which was almost 10% of all known deaths. Mesothelioma rate, both pleural and peritoneal, increased with time since first exposure and appeared to reach a plateau after about 40 to 50 years. The mesothelioma rate increased with amount of exposure and the peritoneal mesotheliomas occurred preferentially in the highest exposure group, 37% compared with 15% overall.

**Conclusion:**

By the end of 2008, the number of mesothelioma deaths had reached 4.7% for all the male workers and 3.1% for the females. Over the past 8 years the numbers were higher than expected. It is predicted that about another 60 to 70 deaths with mesothelioma may occur in men by 2020.

The mortality to 1977 and other health effects of miners and millers employed at the Wittenoom crocidolite asbestos mine in Western Australia was first published in 1980 (Hobbs *et al*, 1980). The mortality up to 2000 from all causes was given by Musk *et al* (2008), and for mesotheliomas by Berry *et al* (2004). In this paper we present mesothelioma incidence and mortality up to the end of 2008, and compared the observed numbers from 1987 to 2008 with predictions that were made from the number occurring up to 1986 (de Klerk *et al*, 1989b; Berry, 1991).

## Subjects and Methods

Crocidolite (blue asbestos) was mined at Wittenoom Gorge in Western Australia from 1937 until 1966. From 1943 a single company (the Australian Blue Asbestos Company) was involved and details of employees of this company were obtained from employment records supplemented by information from a mineworkers’ benevolent fund (Armstrong *et al*, 1988). Characteristics of the workforce have been reported previously (Armstrong *et al*, 1988). Duration of employment was usually short, with 74% of the men and women working at Wittenoom for less than a year and 5% remaining for 5 years or longer. Dust concentrations, particularly in the old mill, were high; only one study of airborne levels was ever carried out and the estimated time-weighted fibre concentrations derived from the results were described as underestimates of exposure by the person who performed the original study (Rogers and Major, 2002), but cumulative exposures calculated from them have been shown to be internally valid based on association with fibre lung burden (de Klerk *et al*, 1996). Almost 90% of the men worked in the mine or mill, or both, but most women did not work in the mine or mill or on the site of operations, but in the town, and consequently their exposures were lower. Women who worked onsite tended to work in the company office which was located within 1 km of the mill (Reid *et al*, 2008).

This analysis is of 6489 men and 419 women; these numbers differ slightly from those reported previously (6493 men and 415 women (Berry *et al*, 2004)) as the continuing follow-up produced revisions to the data previously held. Mortality was assessed by linking the cohort to the National Death Index through the Australian Institute for Health and Welfare (AIHW) and the Western Australian Registrar General for births, deaths and marriages. Cancer incidence was obtained from the National Cancer Statistics Clearing House through the AIHW, the Australian Mesothelioma Registry and the Western Australian Cancer Registry, including the Western Australian Mesothelioma Registry. The largest number of migrants from non English-speaking countries working at Wittenoom was from Italy. There were 1047 workers (16 women) with Italian names and recorded as born in Italy. An unknown number returned to Italy after finishing work at Wittenoom. Italian subjects untraced in Australia were searched in Italy, and their vital status assessed at 31 December 2007. The clinical records of those who died from primary pleural and peritoneal tumours allowed the identification of mesotheliomas (Merler *et al*, 2000). Overall, 954 (91%) of the Italian workers were traced (304 in Italy) including 632 deaths (181 in Italy). The follow-up was based on matching of deaths and cancer notifications, but not all those for whom a match was not found are necessarily still alive and free of cancer. The accuracy of matching of mesotheliomas occurring with Western Australia is probably high as all cases in Western Australia are subjected to detailed investigation by a Mesothelioma Registry Committee, and many are referred to the Sir Charles Gairdner Hospital where a full occupational history is taken. By the end of 2008, 3053 (47%) of the men and 157 (38%) of the women were known to have died.

### Statistical methods

Mesothelioma rates (per 100 000 person years) were calculated by dividing the number of mesotheliomas by person-years at risk. As in our previous follow-up studies the person-years for estimating expected mesotheliomas were counted in two ways: first by assuming that all subjects who were lost to follow-up were still alive at 31 December 2008 (Method 1), and second after censoring all subjects at the date last known to be alive (Method 2). In both cases subjects were censored at age 85 years if necessary. It was necessary to use both methods as it is impossible to obtain a confirmation of ‘still alive’ from official records in Australia. About 2500 of the men responded to a smoking questionnaire first issued in 1979 (de Klerk *et al*, 1991), and repeated at intervals since, and in a study of retinol supplementation that started in 1990 (Alfonso *et al*, 2010), so that there is a recent information on the date last known to be alive for many in this group. The rates calculated with Method 1 are under estimates as there will be some deaths occurring before the age of 85, which have not been discovered through death searches. Conversely, the rates from Method 2 will be overestimated, because subjects have survived for an unknown time after the last date known to be alive.

Mesothelioma rates were analysed with respect to time since first exposure, excluding the first 10 years, subdivided into 5-year intervals, and to cumulative exposure divided into three groups, <10, 10–<50 and >50 fibres per ml years. As noted above, although the absolute level of cumulative exposure may be uncertain, the values have been shown to have internal validity. The mesothelioma rates in the three exposure groups were adjusted for length of follow-up using the indirect method of standardisation.

It was unnecessary to treat exposure as a time-dependent variable because of the short duration of work at Wittenoom for the majority of the subjects. Only 1.2% of the subjects worked at Wittenoom for more than 10 years, and while they were at Wittenoom they contributed only 0.2% of the person-years of follow-up more than 10 years after first exposure.

Tests of significance included the *χ*^2^ test for contingency tables and the Cochran–Armitage trend test. All *P*-values are two-sided.

## Results

The first mesothelioma was notified in November 1960 and the second in October 1969. Thereafter, mesotheliomas were notified regularly and up to the end of 2008 there had been 316 cases in men (268 pleural and 48 peritoneal), and 13 in women (all pleural) (Table 1). The first death with a mesothelioma was recorded in 1961, the second in 1969 and up to the end of 2008 there had been 302 deaths in men with mesotheliomas (255 pleural and 47 peritoneal), and 13 in women (all pleural) (Table 1), so that almost 10% of the known deaths in men, and 8% in women, were due to mesothelioma.

The shortest lag time to diagnosis was 12 years and 9 months (Table 2) and to death 13 years and 5 months, and the longest to diagnosis so far recorded is 58 years. The average lag time to diagnosis was 35.4 years and to death 35.9 years but because the mine and mill closed over 40 years ago, this average and the age of survivors will inevitably rise as follow-up continues, as also will the numbers in the categories of 40 years or more in Table 2.

**Figure 1:**
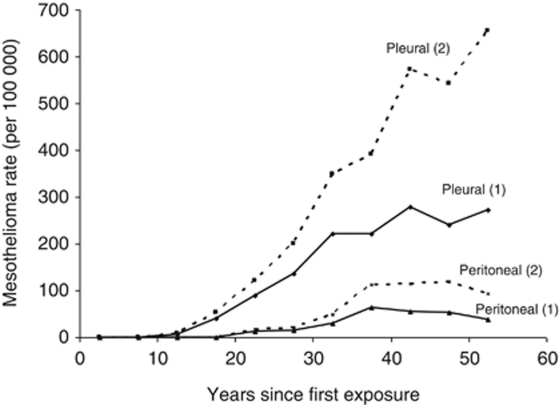
Observed pleural and peritoneal mesothelioma rates by time since first exposed (using Methods 1 and 2 for determining follow-up date).

As expected, the mesothelioma rate increased with time since exposure to both pleural and peritoneal mesotheliomas (Figure 1). A total of 15 pleural mesotheliomas occurred within 20 years of first exposure at Wittenoom but there were no peritoneal mesotheliomas within that time (Table 2). For longer periods of follow-up the proportion of mesotheliomas of peritoneal origin showed no trend with time since the start of exposure (trend *χ*^2^=0.96, 1 DF, *P*=0.33). The mesothelioma rate could only be estimated between the limits of methods 1 and 2 because of the unknown vital status of some subjects (see Methods). Subject to this limitation, the mesothelioma rate appeared to reach a plateau for both pleural and peritoneal mesotheliomas after about 40 to 50 years since first exposure (Figure 1). In absolute terms the number of mesotheliomas was approximately constant between 1986 and 2005 (Table 1).

The mesothelioma rate increased through the increasing exposure categories. For exposure >50 fibres per ml years compared with <10 fibres per ml years, the increase was by a factor of about 5-fold using method 1 for determining follow-up date and about 4-fold using method 2 (Table 3). The majority of mesotheliomas were pleural but 15% were peritoneal, and this proportion varied with exposure, with 37% of mesotheliomas in the highest exposure group being peritoneal, compared with 6% and 7% in the two lower exposure groups (*χ*^2^=46.29, 2 DF, *P*<0.001).

A total of 15% of the pleural mesotheliomas occurred within 25 years of first exposure (Table 3), and over the three exposure groups the percentages were 11%, 17% and 18% (*χ*^2^=2.68, 2 DF, *P*=0.26).

**Figure 2:**
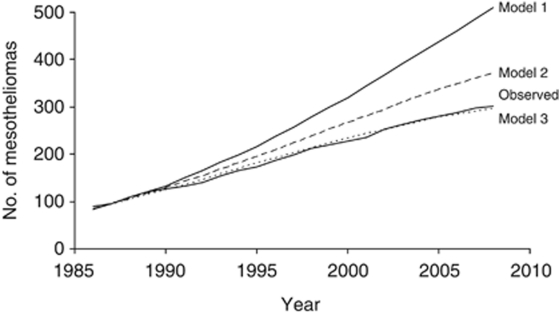
Predicted cumulative number of mesothelioma deaths in men from 1987 to 2008 under three models, and observed number. The observed numbers are close to Model 3 and are identified by a continuous line (observed) and a broken line (Model 3).

On the basis of an analysis of 84 deaths with mesothelioma in men to the end of 1986, predictions were made for the number of occurrence by 2020 (Berry, 1991). The general model used for the mesothelioma death rate in relation to time since first exposure was 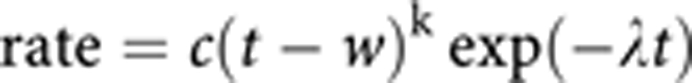 where *c* is related to cumulative exposure, *t* is time since first exposure, k is a power, *w* is a lag time following exposure during which it was assumed that mesotheliomas would not occur, and *λ* is a rate of elimination of crocidolite from the lungs. Five cases of this model were used originally to give a range of predictions, between 339 and 738 mesothelioma deaths in men by 2020. In this paper, attention is restricted to three of the models, the simplest power relationship with no lag period or elimination term (Model 1, *k*=3.5, *w*=0, *λ*=0), and two models allowing the elimination (Model 2, *k*=3.9, *w*=5 years, *λ*=6.8%/year; Model 3, *k*=5.4, *w*=5 years, *λ*=15%/year). Up to the end of 2008, the observed number of deaths fitted closely to the model with the lowest predictions (Table 4), and this model had the elimination coefficient (*λ*) of 15% per year. In Figure 2 the observed and predicted cumulative numbers of mesothelioma deaths are plotted against calendar year to the end of 2008. The observed numbers have continued to fit closely to the predictions based on Model 3, but in the past 8 years the observed number of 74 exceeded the predicted value by 11, suggesting that the numbers are not tailing off as much as predicted.

## Discussion

By the end of 2008 the number of mesothelioma deaths had reached 4.7% for all the male workers and 3.1% for the females. 10% of known deaths in men and 8% in women were due to mesothelioma. The mesothelioma rate increased with time since exposure but seems to have reached a plateau after about 40 to 50 years. This was the case for both pleural and peritoneal mesotheliomas in contrast to the finding in an Italian asbestos cement plant of a continuing increase in the rate of peritoneal mesotheliomas after a plateau was reached for pleural mesotheliomas after about 40 years (Barone-Adesi *et al*, 2008; Magnani *et al*, 2008). The mesothelioma rate increased with cumulative exposure. Overall, 15% of mesotheliomas were peritoneal and the proportion of mesotheliomas that were peritoneal was highest (37%) in the highest cumulative exposure group. This is in accordance with the concept suggested by Selikoff *et al* (1970) over 40 years ago that pleural mesotheliomas are associated with lesser exposure and peritoneal with greater, and with the meta-analysis of Hodgson and Darnton (2000).

The increasing mesothelioma rate with cumulative exposure implies that the mesothelioma rate reaches a particular level within fewer years after first exposure for the more highly exposed. As an example, the pleural rate reaches 200 per 100 000 between 32 and 42 years after exposure in the lowest exposure group, after about 26 years in the next exposure group, and after 21 years for the highest exposure group. However, the shapes of the relationship between mesothelioma rate and time since exposure were similar for the three exposure groups, and the percentages of mesotheliomas occurring within the first 25 years after exposure were also similar, suggesting that the pattern over time of latency was in relative terms independent of exposure. This is in general agreement with the findings of Hansen *et al* (1998) in Wittenoom residents and earlier results in the workers (de Klerk *et al*, 1989a).

By 2000 the models based on the number of deaths with mesothelioma up to 1986 gave predictions of around 234–320 mesothelioma deaths in men and the observed number was 224 (Berry *et al*, 2004) so that it was clear that model with the lowest predictions (Model 3) was the best fitting. This is now strengthened by the finding that the number of deaths with mesothelioma in men in the period 1987 to 2008 remained similar to the lowest predictions and this is an evidence that models of mesothelioma incidence that incorporate an allowance for a gradual elimination of crocidolite from the lungs after exposure are more realistic. There is evidence that elimination of crocidolite asbestos occurs (Berry, 1999), and from regression analysis of post mortem lung content of crocidolite fibres on years since last exposure in 90 former Wittenoom workers, a clearance rate of 9% a year was estimated after allowing for exposure (de Klerk *et al*, 1996). The best fitting prediction model had a higher rate of 15% suggesting that there may be factors, in addition to actual elimination, operating to attenuate the rate at longer periods of follow-up.

The number of mesotheliomas in the past 8 years was higher than predicted, 74 observed were compared with 63 predicted, and if this continues the cumulative number by 2020 would be >339 as given by the best fitting predictive model. Also the predictions were based on 84 mesotheliomas by 1986 but with later information this has become 89, and allowing for this increase the predicted number by 2020 may be 359. This implies that there may be about a further 60 to 70 deaths in men due to mesothelioma by the end of 2020. The toll from the Wittenoom mine and mill, which closed over 40 years ago will continue for more than another 10 years.

## Figures and Tables

**Table 1:** Diagnosed and deaths^a^ with mesotheliomas by period

	**Men**			**Women**	
**Period**	**Pleural**	**Peritoneal**	**Total**	**All pleural**	**Total**
1960–65	1/1	0/0	1/1	0/0	1/1
1966–70	3/3	0/0	3/3	0/0	3/3
1971–75	13/10	0/0	13/10	0/0	13/10
1976–80	23/18	5/4	28/22	1/1	29/23
1981–85	36/32	5/5	41/37	1/1	42/38
1986–90	42/47	7/7	49/54	2/1	51/55
1991–95	43/41	6/6	49/47	3/3	52/50
1996–00	44/42	12/12	56/54	0/1	56/55
2001–05	38/42	11/10	49/52	4/3	53/55
2006–08	25/19	2/3	27/22	2/3	29/25
Total	268/255	48/47	316/302	13/13	329/315

a Numbers in each cell are diagnosed per deaths. Within the cells the number of deaths are not a strict subset of the diagnosed as in some cases death occurred in a later period than diagnosis.

**Table 2:** Lag time between starting at Wittenoom and diagnosis with mesothelioma

	**Men**			**Women**	
**Lag (years)**	**Pleural**	**Peritoneal**	**Total**	**All pleural**	**Total**
11–15	2	0	2	0	2
15–19	13	0	13	0	13
20–24	26	4	30	1	31
25–29	37	4	41	2	43
30–34	55	8	63	3	66
35–39	50	15	65	4	69
40–44	49	10	59	1	60
45–49	23	5	28	1	29
50–54	9	1	10	1	11
55–59	4	1	5	0	5
Total	268	48	316	13	329

**Table 3:** Mesotheliomas by cumulative exposure

		**Cumulative occupational exposure (f per ml years)^a^**		
	**Total**	**<10**	**10–<50**	**⩾50**
Number of men	6484	4120	1623	736
Number of women	419	397	20	1
				
*Number of diagnosed mesotheliomas*				
Men				
Pleural	268	112	99	55
Peritoneal	48	8	8	32
Women				
Pleural	13	9	4	0
Total	329	129	111	87
				
*Mesothelioma rate*^b^ *per 100 000 person-years*^c^				
Pleural+peritoneal				
Method 1 (adjusted^d^)	158	93 (92)	225 (226)	442 (471)
Method 2 (adjusted)	238	146 (144)	324 (327)	553 (581)
Pleural				
Method 1 (adjusted)	135	87 (86)	208 (209)	280 (297)
Method 2 (adjusted)	203	137 (136)	300 (303)	349 (367)
Peritoneal				
Method 1 (adjusted)	23	6 (6)	17 (17)	163 (176)
Method 2 (adjusted)	35	9 (9)	24 (24)	203 (216)
Pleural mesotheliomas within 25 years of first exposure (% of total)	42 (15%)	13 (11%)	18 (17%)	10 (18%)

a Cumulative occupational exposure was unknown for six subjects including two with mesothelioma.

b Mesothelioma rates calculated excluding the first 10 years of follow-up.

c Three mesotheliomas occurring after the age of 85 years were excluded from this analysis.

d Standardised for length of follow-up using the indirect method.

**Table 4:** Predicted and observed number of mesothelioma deaths in men 1987–2008

	**Predicted (cumulative)**			
**By end of the year**	**Model 1, k=3.5, w=0, λ=0**	**Model 2, k=3.9, w=5 years, λ=6.8% per year**	**Model 3, k=5.4, w=5 years, λ=15% per year**	**Observed**
1986	84^a^	84^a^	84^a^	89^b^
1990	133	128	124	127^c^
1995	216	194	180	174^c^
2000	320	267	234	228^c^
2008	509	371	297	302

a Observed number as known at the time of prediction.

b Observed number revised.

c These figures were 126, 173 and 224 in 2004 paper.
